# Direct marketing in health and medicine: using direct mail, email marketing, and related communicative methods to engage patients

**DOI:** 10.1186/s12913-020-05603-w

**Published:** 2020-09-15

**Authors:** James K. Elrod, John L. Fortenberry

**Affiliations:** 1 Willis-Knighton Health System, 2600 Greenwood Road, Shreveport, LA 71103 USA; 2 LSU Shreveport, 1 University Place, Shreveport, LA 71115 USA

**Keywords:** Direct marketing, Marketing communications, Promotion, Hospitals, Healthcare

## Abstract

**Background:**

Direct marketing—the delivery of messages via mail, the Internet, and similar routes directly to consumers—is used extensively by healthcare organizations to attract and inform current and prospective patients of health and medical offerings and opportunities. Examples of direct marketing include direct-mail marketing, telemarketing, and Internet marketing, with routes being selected on the basis of their ability to reach desired audiences. The various avenues offered by direct marketing afford options to address most any sought group.

**Discussion:**

Direct marketing is one of the most recognized forms of marketing communication, thanks in large part to its widespread use and direct engagement of consumers. While some applications clearly have the potential to irritate consumers (e.g., junk mail in post boxes, spam in email inboxes), direct marketing can be deployed in manners respectful of recipients and, in such cases, it can prove to be a helpful communications asset. To aid others in understanding this particular conveyance method, this article presents an overview of direct marketing and shares deployment insights and experiences from Willis-Knighton Health System.

**Conclusions:**

Direct marketing provides a useful communications pathway, permitting health and medical institutions to educate and enlighten desired audiences. Given instances of overuse and misuse by organizations, however, great care must be taken to design and deploy direct marketing initiatives inoffensively. If well designed and respectfully implemented, direct marketing affords significant communications utility, earning a valued place in the marketing communications arsenals of healthcare establishments.

## Background

Patient acquisition and retention activities constitute some of the most important tasks conducted by health and medical establishments [1–6]. Without consistent and, ideally, growing patient volume, institutional viability becomes uncertain, potentially threatening the very existence of given medical providers and, in turn, the livelihoods of their employees and the health status of the people they serve [1, 7–9]. Seen in this light, inabilities to attract and retain patients carry consequences well beyond the walls of given healthcare establishments, extending deeply into markets and even impacting community health [10, 11]. This fact, of course, provides significant motivation, compelling many healthcare providers to direct intensive efforts toward ensuring that ongoing streams of patients are secured, fostering viability that affords mutual benefits [1, 7].

In pursuing viable patient streams, communication proficiencies are essential, affording health and medical establishments with the ability to engage, inform, and attract current and prospective customers. Ultimately, this yields all-important patient volume and institutional market share [3, 5, 12, 13]. Pathways abound for reaching desired audiences, with one in particular, known as direct marketing, effectively delivering messages from given healthcare institutions directly to sought audiences, typically via mail, telephone, or Internet communications tools [1, 7]. Direct marketing is heavily utilized by health services organizations, with applications ranging from postcards mailed to prospects which introduce newly available medical technologies to emails which invite recipients to attend the open houses of given healthcare establishments [1, 5–8].

Direct marketing is one of the most recognized forms of marketing communication, thanks in large part to its widespread use and direct engagement of consumers. While some applications clearly have the potential to irritate consumers (e.g., junk mail in post boxes, spam in email inboxes), direct marketing can be deployed in manners respectful of recipients and, in such cases, it can prove to be a helpful communications asset [14–16]. To aid others in understanding this particular conveyance method, this article presents an overview of direct marketing and shares deployment insights and experiences from Willis-Knighton Health System.

## Definition and overview

Direct marketing is one of many elements constituting the broad discipline of marketing, formally defined as “a management process that involves the assessment of customer wants and needs, and the performance of all activities associated with the development, pricing, provision, and promotion of product solutions that satisfy those wants and needs” [1], p. 288. Promotion, as evidenced in this definition, is a core feature of marketing, earning inclusion as one of the Ps in the classic expression known as the *four Ps of marketing* (i.e., Product, Price, Place, Promotion). The promotion aspect of marketing essentially entails any and all elements associated with engaging audiences, with the core pathways for engagement being depicted in a descriptive model known as the marketing communications (or promotions) mix [1, 17].

Classically illustrated, the marketing communications mix contains five principal avenues of communication; namely, advertising (i.e., the paid use of mass media to deliver messages), personal selling (i.e., the use of sales agents to personally deliver messages), sales promotion (i.e., the use of incentives, such as contests and free giveaways, to encourage patronage), public relations (i.e., the use of publicity and other unpaid promotional methods to deliver messages), and direct marketing (i.e., the delivery of messages via mail, the Internet, and similar routes directly to consumers) [1, 7]. Healthcare providers examine each of these communicative avenues, selecting one or more believed to be most capable of reaching target audiences, with the ultimate goal being to encourage patronage or compel some other desired action [1, 9].

Direct marketing is characterized by its conveyance of information directly to individuals. Unlike advertising, which uses mass media to deliver messages to broad audiences en masse, hoping to entice interested parties into some form of desired exchange, direct marketing engages individuals directly by sending, for example, a promotional brochure, email message, or similar conveyance straight to the intended recipient. It is a highly targeted form of communication and, as such, is highly measurable, as responses to various solicitations can be tracked with relative ease [14–16, 18]. Historically, direct marketing often brought to mind telemarketing or direct mail, but times have changed. Today, telemarketing has been deemed by society to be so undesirable that its use is now highly regulated, diminishing opportunities and associated presence considerably. Even though direct mail often is characterized by recipients as junk mail, it continues to be used quite heavily, although shifts to electronic forms of communication have diminished its popularity. The Internet indeed has ushered in numerous direct marketing opportunities, ranging from email appeals to newsletter distribution to social media engagements. This particular avenue is evolving rapidly and almost certainly represents the future of direct marketing methods [1, 14, 16].

One of the most critical tasks associated with direct marketing pertains to building lists of prospects who will be targeted with associated solicitations [5, 14, 15]. Prospect lists often are purchased from vendors who specialize in the provision of such, permitting healthcare establishments to designate recipient characteristics (e.g., ZIP code, gender, age, interests, etc.) and request lists of candidates meeting associated criteria. Although more labor intensive, health and medical institutions can opt instead to build their own lists. Assembling these lists typically begins by asking current customers if they would like to receive collateral, such as monthly newsletters, invitations to special events, promotional messages detailing new healthcare options, and so on, adding those desirous of such to a direct marketing database. Invitations to join mailing lists, subscribe to social media news conveyances, and the like also can be inserted into other marketing communications, with those opting in being added to associated direct marketing repositories. With concerted efforts over time, custom lists will grow and become true institutional assets, typically exceeding the value derived from their more generic, purchased counterparts [5, 7, 14–21].

How lists are used arguably is just as important as quality of given lists. Simply collecting contact details and sending solicitations whenever healthcare institutions please is decidedly poor form and likely will engender the animosity of recipients. This practice historically has been used by many organizations and unfortunately continues to this day, perpetuating negative feelings regarding direct marketing [5, 7, 14–21]. Such animosity can be reduced or eliminated entirely by practicing what is known as permission marketing, requiring that institutions request and be granted permission before forwarding solicitations to intended recipients and, for those granting permission, offering easy methods to opt out of future solicitations [16, 22].

Delivery without permission to do so can damage institutional reputations and lead to senders being labeled “junk mailers,” “spammers,” and the like for directing unsolicited and often unwanted correspondence to individuals. As tempting as it might be to send promotional messages without an invitation, it should be avoided, as the main aim of healthcare communications rests with establishing a productive dialogue, a mission immediately destroyed by intruding on the personal space of audiences [5–7, 14–22]. Of course, beyond the assembly of lists and their proper use, the information contained in direct marketing pieces must have value to recipients. If healthcare providers develop direct marketing programs that fulfill these mandates, they can expect good things from their associated programs.

## Institutional background, deployment history, and context within marketing communications

From its earliest of days, dating back to 1924, Willis-Knighton Health System has emphasized communications excellence, something which in present times remains a strategic priority, compelling extensive communicative experimentation and innovation. Headquartered in Shreveport, Louisiana and situated in the heart of an area known as the Ark-La-Tex where the states of Arkansas, Louisiana, and Texas converge, Willis-Knighton Health System holds market leadership in its served region where it delivers comprehensive health and wellness services through multiple hospitals, numerous general and specialty medical clinics, an all-inclusive retirement community, and more. The institution’s achievement of market leadership is attributed, in part, to communications prowess, permitting Willis-Knighton Health System to effectively engage current and prospective patients, evoking interest and attention, ultimately leading to burgeoning patient volume and customer loyalty.

Today, Willis-Knighton Health System leverages the power of the full marketing communications mix, deploying all of its components, including direct marketing. The establishment’s use of direct marketing has been fairly consistent over its history, best characterized as a modest deployment, primarily used to complement other forms of marketing communication. Its use in this manner stems simply from Willis-Knighton Health System’s preferences for other forms of marketing communication which, based on the institution’s experiences, are more effective at achieving its designated communications goals. For direct marketing’s part, associated applications deployed by Willis-Knighton Health System have evolved as the particular medium of communication and consumer preferences have advanced over time.

**Fig. 1:**
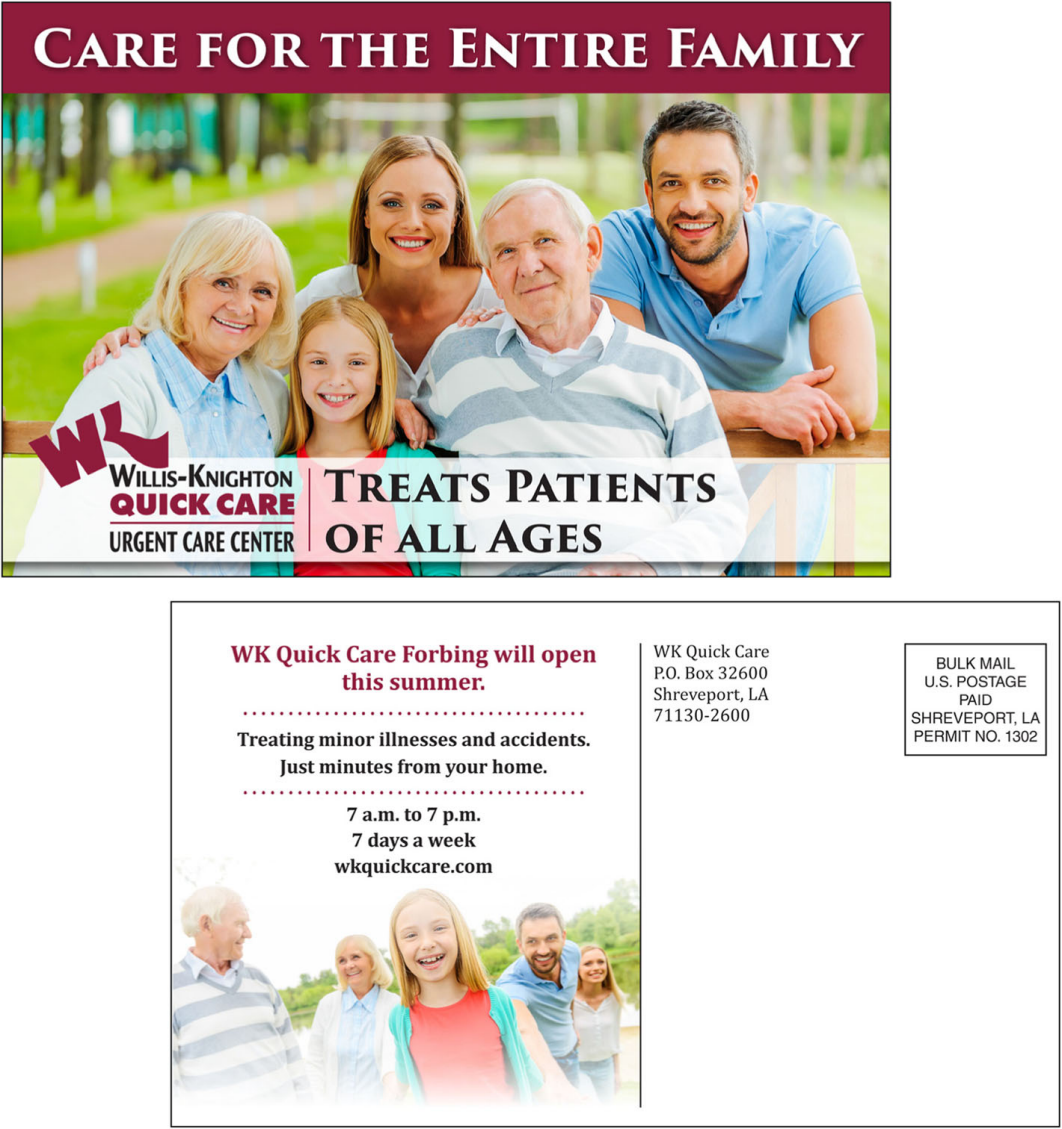
A postcard promoting Willis-Knighton Health System’s Quick Care Urgent Care Center

Of major forms of direct marketing, telemarketing, which entails contacting desired audiences via telephone, historically has been used sparingly by Willis-Knighton Health System due to the practice’s disruptive nature. In recent decades, direct mail, which entails sending solicitations via post, has accounted for the majority of direct marketing deployments by the institution, with the typical application being in the form of postcards introducing new physicians, communicating new services, and so on to inform and enlighten recipients, as demonstrated by the example presented in Fig. 1. Willis-Knighton Health System’s most prominent direct mail effort is the institution’s healthy lifestyles magazine known as *Vim & Vigor* [23]. This magazine features a variety of health and wellness stories, along with helpful details about Willis-Knighton Health System and its associated services. *Vim & Vigor* is mailed to individuals and institutions throughout the Ark-La-Tex region and serves as a helpful vehicle for building and maintaining awareness.

Postal distribution of *Vim & Vigor* is complemented by electronic distribution via Willis-Knighton Health System’s website, with recent issues being viewable at the following link: https://www.wkhs.com/about/vim-vigor. This move was inspired by very clear trends which indicate increasing use of and preferences for Internet-based communications by consumers. This particular avenue of communication is capturing an expanding share of the institution’s direct marketing dollar as paper-based and posted methods fall out of favor as Internet communications continue to develop and proliferate [3]. In keeping with this trend, Willis-Knighton Health System has directed more attention toward electronically-distributed direct marketing applications, with the most notable initiatives being email-directed communications and subscriptions to social media platforms, following a permission marketing protocol.

Although occupying a relatively small portion of Willis-Knighton Health System’s overall marketing communications budget, direct marketing is fulfilling its designated purpose, complementing other marketing communications deployed by the institution. The medium, of course, is capable of more robust deployments, with the method and manner of given applications being determined based on the wants and needs of particular establishments. Regardless of approach and intensity, care must be taken to ensure that deployments are respectful of recipients, something that is essential in order for direct marketing to fulfill its intended purpose of informing and enlightening recipients.

## Strengths

Willis-Knighton Health System’s observations from its direct marketing experiences suggest a number of strengths, compelling the avenue’s inclusion in the institution’s marketing communications mix composition. Chief strengths of direct marketing are described as follows.

### Ability to be precisely targeted

As suggested by its name, direct marketing is forwarded directly to individual recipients, affording precise targeting which permits personalization and minimizes wasted circulation [5, 6, 14–21]. Something as simple as addressing a parcel using a person’s actual name, as opposed to “Resident” or something equally nondescript, can improve the chances that the parcel will be noticed. This is all the more the case for more robust forms of customization, such as inserts which reference the recipient by name, and, with permission marketing elements in place, forwarding information for which the individual has already expressed an interest in receiving. Without such customization, direct marketing applications are more likely to be disregarded, constituting circulation which delivers no value. Since the goal of direct marketing is to engage, the investment required to compel someone to actually look at pieces received makes perfect sense. Doing the opposite and deciding to simply send generic parcels will foster inattentiveness and yield a diminished return on investment. As such, healthcare institutions engaged in direct marketing should take advantage of the communicative method’s precision targeting capability and customize conveyances, accordingly.

### Highly measurable performance

Unlike many forms of marketing communication, direct marketing happens to be highly measurable, something enhanced further by particular creative applications, permitting establishments to better determine return on investment [14–21]. Direct mail pieces, for example, can feature a particular telephone number in the given message, providing reasonable assurances that calls directed to that number were generated by the noted direct mail campaign. A newsletter distributed to recipients which conveys a special offer that is only promoted in the given communication provides similar opportunities to ascertain impact by tying results to the particular direct marketing campaign. Direct marketing applications placed via the Internet permit a wealth of tracking opportunities through such things as the inclusion of web links tied to given campaigns, the ability to access data analytics details, including recipient click behaviors, and more, giving perhaps the greatest utility for assessing campaign results. Such measurability aids in shaping and honing approaches by reviewing experiences of prior campaigns, making adjustments as needed for improvements in future campaigns. This also is most helpful in determining—and justifying—marketing communications budgets.

### Potential to convey significant information

Direct marketing efforts have the potential to telegraph significant amounts of information [5, 6, 14–21]. Whereas a 30-second television commercial can only effectively convey a limited amount of detail, direct marketing, courtesy of its delivery methods, can feature a wide range of information. This is especially helpful in the health services industry where offerings typically are highly complex, necessitating robust details in order for consumers to make informed decisions. A direct mail parcel posted to residents might, for example, contain a multipage brochure, information booklet, or other form of collateral, conveying extensive facts and figures which can be consumed at the leisure of recipients. Internet communications offer the same potential, including the convenience of featuring helpful web links which can be directly accessed by the recipients of given communications. This particular attribute actually works quite well for reinforcing other forms of marketing communication, with advertising building top-of-mind awareness for, say, a given medical procedure, and a complementary direct marketing campaign delivering enhanced information to bolster awareness of the given innovation, yielding effective marketing communications synergies.

## Limitations

Motivations to use direct marketing are counterbalanced by a series of limitations which must be factored into applications so as to minimize or avoid associated effects. Notable limitations are described as follows.

### Potential for intrusiveness

Direct marketing, courtesy of its direct engagement attribute, has the potential to intrude on the privacy of recipients, something which is magnified when organizations carelessly and selfishly deploy the medium of communication [5, 6, 14–21]. Many have experienced, for example, unrelenting telephone solicitations, junk mail cluttering mailboxes and hampering one’s ability to locate meaningful parcels, and spam messages crowding email inboxes and diminishing associated utility. Such nuisances have harmed the reputation of direct marketing, necessitating extreme care in its deployment. As described in prior sections, the potential for intrusiveness can be diminished or eliminated entirely with the establishment of a protocol which requires receipt of permission prior to forwarding direct marketing communications to targets. Doing so eliminates wasted circulation and ensures that direct marketing efforts are not reputationally damaging by overstepping the boundaries of recipients.

### Potential to be overlooked

The volume of direct marketing efforts forwarded to consumers generally is staggering, overwhelming recipients who often will not take the time to separate meaningful and relevant communications from the many which are not. The end result of this proliferation is consumer inattentiveness to direct marketing appeals [5, 14–21]. This, of course, makes it very difficult for institutions forwarding targeted, relevant, and respectful direct marketing communications to break through the clutter to win the attention of recipients. Stories of people visiting their mailboxes, grabbing parcels, and disposing of junk mail without as much as a second glance abound, as do accounts of individuals opening their email inboxes and deleting messages en masse, not wishing to take the time to screen the sea of solicitations often flooding their accounts. Breaking through the clutter is challenging for anyone engaged in direct marketing, with perhaps the best method for doing so being through the deployment of highly creative applications which capture attention. These, of course, must be infused with communications which are relevant and delivered respectfully.

### Database management challenges

As noted in prior sections of this article, the success of direct marketing campaigns is heavily reliant on the quality of the recipient list. Quality must extend beyond correct contact details, reaching into deeper things like communication preferences (e.g., mail, telephone, email), content desired (e.g., promotional messages from the host organization, promotional messages from partner organizations), relevance to the recipient (e.g., sports medicine services for athletically-inclined individuals, senior-related health services for senior citizens), and the like. These details must be managed properly and updated in a timely fashion, with this exercise representing a significant challenge [5, 14–21, 24]. Proper database management also is essential for ensuring that direct marketing permissions, which can and do change often, are accurate. Those opting in or opting out must be registered as such immediately as part of prudent efforts to ensure that audiences are addressed as they desire. Resources obviously are required for database management endeavors, ranging from information systems to personnel charged with overseeing and effecting processes. Despite associated challenges, such investments have the potential to dramatically improve direct marketing efforts.

## Operational reflections

For administering any component of the marketing communications mix, Willis-Knighton Health System advises establishing a baseline foundation of resources, including (1) top leadership support and commitment, (2) financial resources sufficient for funding communications activities, (3) competent personnel charged with effecting given initiatives, and (4) formal processes permitting effective planning, implementation, and evaluation of initiatives. Adequate resources set the stage for productive audience engagement endeavors, minimizing chances of resource-depleting and reputation-damaging mistakes which, in the realm of marketing communications, often are very public, given the open circulation of such conveyances. These resources also ensure competencies in using given marketing communications mix components, with proper deployment being essential for realizing desired outcomes.

Beyond the advisories conveyed elsewhere in this article, Willis-Knighton Health System suggests that health and medical establishments considering the use of direct marketing make certain that they carefully consider the total costs of the particular applications under examination. Minimally, healthcare entities will need to consider the costs associated with (1) development of creative content, (2) production of associated collateral, such as printing in the case of direct mail, (3) purchasing or building a recipient list, including database management expenses, (4) distribution fees—such as postage in the case of direct mail—associated with forwarding direct marketing communications to recipients, and (5) labor costs associated with effecting given campaigns. Once expenditures are tallied, healthcare providers then are positioned to compare the costs of the proposed direct marketing campaign with the costs of other forms of marketing communication, providing a useful evaluative measure for marketing communications planning. Such examinations aid in ensuring that total costs are considered whenever contemplating direct marketing campaigns, permitting health and medical organizations helpful assistance in determining the most prudent avenues available for achieving designated communicative goals.

## Conclusions

Direct marketing provides a useful communications pathway, allowing health and medical institutions to educate and enlighten desired audiences. This sets the stage for acquiring patronage and resulting market share, yielding numerous mutual benefits for given establishments and their communities. As with all forms of marketing communication, care must be taken to deploy direct marketing properly, capitalizing on its strengths while avoiding applications that evoke its limitations. Database assembly and management activities are particularly critical and a permission marketing mindset is imperative for achieving the best results. Further, given instances of overuse and misuse by organizations, great care must be taken to design and deploy direct marketing initiatives inoffensively. If well designed and respectfully implemented, direct marketing affords significant communications utility, earning a valued place in the marketing communications arsenals of health and medical organizations.

## Data Availability

Not applicable.
